# The relationship between parental depression and child internalizing and externalizing problems: The roles of parenting stress and child maltreatment

**DOI:** 10.3389/fpubh.2023.962951

**Published:** 2023-02-07

**Authors:** Chen Chen

**Affiliations:** Center for Educational Science and Technology, Beijing Normal University, Zhuhai, China

**Keywords:** parental depression, child behavior problems, parenting stress, child maltreatment, Chinese samples

## Abstract

**Introduction:**

Although the relationships between parental mental health and child internalizing and externalizing problems have been explored by previous studies, the pathways between these two variables need further exploration. The present study aims to explore the relationships between parental depression and child internalizing and externalizing problems and to examine the roles of parenting stress and child maltreatment in those relationships within the Chinese cultural context.

**Method:**

Data were collected from 855 Chinese families with preschool-aged children, and mediation analysis was used to examine the pathways between these variables.

**Results:**

The results show that parental depression is positively associated with child internalizing and externalizing problems, and child maltreatment and the combination of parenting stress and child maltreatment mediated the relationships between parental depression and child internalizing and externalizing problems, respectively. These findings suggest that parental depression not only has a direct effect on child internalizing and externalizing problems but also has an indirect effect *via* parenting stress and child maltreatment.

**Discussion:**

Decreasing the levels of parenting stress and child maltreatment should be applied in interventions to break the relationships between parental depression and child internalizing and externalizing problems within the Chinese cultural context.

## Introduction

Internalizing and externalizing problems, including maladaptation in emotions and behaviors, are important issues in child development, particularly in children of preschool age (1), but they also have long-term effects on later behavioral, emotional, and social development (2, 3). Approximately 20% of Chinese preschool-aged children have internalizing and externalizing problems (4), and several factors increase the risk of these problems, such as parental depression (5). Parents with high levels of depression may increase the levels of internalizing and externalizing problems in children (6). However, there is little knowledge about the pathways between these two variables. Parents who have high levels of depression may pay attention to negative events (7), which may increase their parenting stress, and these high levels of parenting stress may increase the risk of child maltreatment (8), which, in turn, contributes to child internalizing and externalizing problems (9). According to Goodman and Gotlib (10), the context, particularly the stressors, of the lives of children in families with depressed mothers, contributes significantly to the development of psychopathology in children. Guided by this notion and by the family system theory that emphasizes that the family is a complex emotional unit and family members influence each other (11), the current study attempts to verify the relationships between parental depression and child internalizing and externalizing problems and to examine the mediating factors (e.g., parenting stress and child maltreatment) of those relationships.

## Parental depression, internalizing and externalizing problems, and parenting stress

A growing body of research has shown that parental depression is positively associated with children internalizing and externalizing problems. Based on a cross-sectional study with 2222 Chinese parents, Ma et al. (12) reported that parental depression was positively associated with internalizing and externalizing problems of preschool-aged children. Zong et al. (13) confirmed these results based on a longitudinal study with Chinese samples. Moreover, Marçal (14) found that parental depression positively predicted child internalizing and externalizing problems based on a longitudinal study with Western samples. Parents with depression may have high levels of marital conflicts (15) and hostile parenting methods (16), contributing to high levels of child internalizing and externalizing problems (17). Additionally, according to the model of relationships between depressed mothers and child development discussed by Goodman and Gotlib (10), depressed parents may cause impairments in their child's later development.

Although large extensive research has confirmed the relationships between parental depression and internalizing and externalizing problems in children across nations and cultures, the pathways between these two variables still need further exploration. Parenting stress, an index for levels of stress in parenthood, might be a factor in the relationships between parental depression and child internalizing and externalizing problems. Parents with high levels of depression may have high levels of parenting stress, namely in new parents (18, 19), and Galbally et al. (20) confirmed these results in a longitudinal study with 246 Australian parents of toddlers. Meanwhile, Salloum et al. (21) reported on these relationships among parents of children aged 8–12 years old. Those relationships have also been confirmed in parents of adolescents with Attention-Deficit-Hyperactivity Disorder (22).

Moreover, parenting stress predicts if a child will later develop internalizing and externalizing problems, as seen in previous studies (23, 24). For example, based on a Chinese cross-sectional study, Li et al. (25) reported that parenting stress was positively associated with internalizing and externalizing problems of 317 preschool-aged children. Based on a longitudinal study, Han amd Lee (26) found that parenting stress positively predicted 1724 Korean preschool-aged children's internalizing and externalizing problems. Meanwhile, also based on a longitudinal study, Kochanova et al. (27) found these relationships in 1209 American children in early childhood, and de Maat et al. (28) also confirmed those relationships based on a study conducted with 441 European adolescents and their parents.

Even though the associations between parental depression, child internalizing and externalizing problems, and parenting stress have been explored by extensive research, few studies have explored the roles of parenting stress in the relationships between parental depression and child internalizing and externalizing problems, particularly in children of preschool age. Parents with high levels of depression may have attention biases to negative information (29), which may increase parenting stress (30), and, finally, contribute to child internalizing and externalizing problems (31). Thus, we hypothesize that parenting stress mediates the relationships between parental depression and child internalizing and externalizing problems within the Chinese cultural context.

## Parental depression, internalizing and externalizing problems, and child maltreatment

Child maltreatment, including negative parenting behaviors, has been explored in extensive studies, and parental depression may be a risk factor for child maltreatment (32). David (33) found that 62% of parents who have maltreated their children had mental health problems (e.g., depression). Based on a longitudinal study with 1,813 families, Mustillo et al. (34) reported that parental depression positively predicted child maltreatment in children aged 0 to 14. Plant et al. (35) found that parental depression alone did not predict child maltreatment, but the combination of parental childhood maltreatment and depression predicted child maltreatment.

Moreover, the relationships between child maltreatment and child internalizing and externalizing problems have been explored by previous studies. Based on a longitudinal study, Isumi et al. (36) found that child maltreatment positively predicted later internalizing and externalizing problems in Japanese children aged 6 to 10. Watters et al. (37) reported that, based on a longitudinal study, child maltreatment positively predicted later internalizing and externalizing problems in 1067 American adolescents. Ma et al. (38) confirmed these results in a study with 2180 South Korean adolescents. However, Godinet et al. (39) did not find a relationship between child maltreatment and internalizing and externalizing problems among preadolescent girls.

Although associations between parental depression, child internalizing and externalizing problems, and child maltreatment have been explored by extensive research, few studies have explored the role of child maltreatment in the relationships between parental depression and child internalizing and externalizing problems. Moreover, depressed parents may cause negative attributions in child behaviors (12), which may increase the risk of negative parenting behaviors, such as maltreatment behaviors. Thus, we hypothesize that child maltreatment mediates the relationships between parental depression and child internalizing and externalizing problems within the Chinese cultural context.

## Parenting stress and child maltreatment

Parenting stress may be a risk factor for child maltreatment (40), and the relationship between these two variables has been explored by previous studies. For example, Crouch et al. (41) found that parents who have high levels of parenting stress had three times the risk of maltreating their children than parents with low levels of parenting stress. Maguire-Jack and Negash (42) reported that parenting stress positively predicted child maltreatment in 1045 American families.

Although previous studies have explored the separate roles of parenting stress and child maltreatment in the relationship between parental characteristics and child development, few studies have examined the roles of the combination of parenting stress and child maltreatment in those relationships. We hypothesize that parenting stress and child maltreatment progressively mediate the relationships between parental depression and child internalizing and externalizing problems.

## The current study

**Figure 1:**
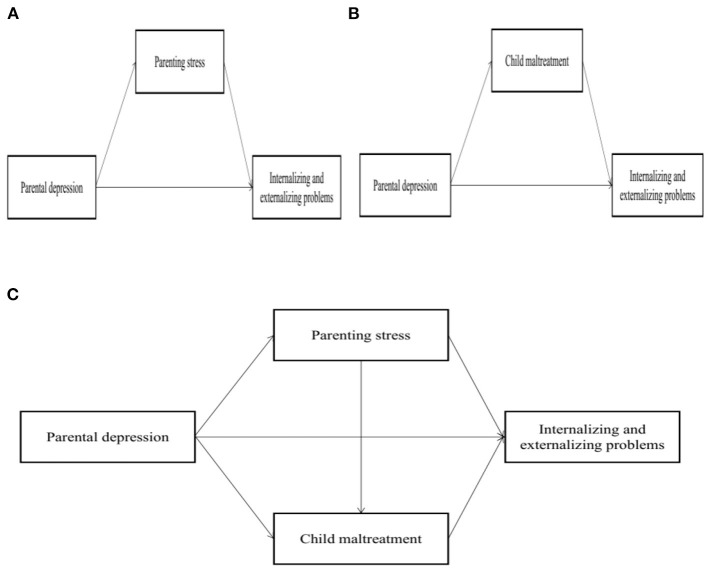
Hypotheses models. **(A)** model: Parenting stress model; **(B)** model: Child maltreatment model; **(C)** model: The combination of parenting stress and child maltreatment model.

Although previous studies have explored the relationships between parental depression and child internalizing and externalizing problems, few studies have examined the roles of parenting stress and child maltreatment in those relationships, particularly in Chinese families with preschool-aged children. China, an ancient Eastern country, has a long history of endorsing Confucian culture, which may influence the values, thinking patterns, and beliefs of the modern Chinese population. Traditional Chinese culture emphasizes the importance of children in families [e.g., Wang Zi Cheng Long highlighted the hope within families that children have a bright future) and harsh discipline for educating children (e.g., physical punishment; (43)], which may increase parenting stress and negative parenting behaviors. Thus, parenting within the Chinese cultural context may be different from Western countries, which may raise the importance of exploring these issues within the Chinese cultural context. Moreover, environmental factors (e.g., COVID-19) may also influence parental mental health, which may increase the risks of parenting stress and child maltreatment, and contribute to maladaptation in child development. Therefore, the current study attempts to verify the relationships between parental depression and child internalizing and externalizing problems and to examine the roles of parenting stress and child maltreatment in Chinese families with preschool-aged children. We hypothesize that parenting stress and child maltreatment play a mediation role in the relationships between parental depression and child internalizing and externalizing problems, respectively, as well as the combination of parenting stress and child maltreatment (see Figure 1).

## Methods

### Participants

The project of ability and nurture in dreaming age (PANDA), a Chinese longitudinal study with a six-month interval, aimed to explore the relationships between external environment factors (e.g., family, school, and community) and individuals' development from preschool to middle school within the Chinese cultural context. The PANDA initially recruited 900 families from five preschools in South China, and 45 families dropped out during the data collection process. The current study used the data of Wave 1 in PANDA, which was collected in May 2021. In 855 families, 15.8% (135/855) of fathers completed the survey, and all of the parents had married within five years of the survey date (3 years: 11.5%; 4 years: 29.9%; 5 years: 58.6%). For family monthly income, 0.8% of the families were below 2500RMB ($368.25), 2.5% fell into the range from 2500RMB to 5000RMB ($736.5), 12.9% fell into the range from 5001RMB to 10,000RMB ($1,473), 56.1% fell into the range from 10,001RMB to 30,000RMB ($4,419), and 27.6% of the families earned above 30,000RMB (the median income in the geographical area of the study was 8,832 RMB). Moreover, the mean children's age was 4.55 years (SD = 0.85), with a range from 3 to 6 years, and 52.2% (446/855) of the children were boys.

### Measures

The Center for Epidemiologic Studies Depression Scale (CES-D) is a self-report scale with 20 items that was developed by Radloff (44) with the aim of assessing levels of depression in daily life. Each item is rated on a four-point Likert scale ranging from “1 = *rarely or*<*1 day*” to “4 = *most of the time*,” and high scores indicate high levels of depressive symptoms. Wang et al. (45) translated and validated the CES-D to the Chinese cultural context, and the Chinese version of the CES-D has been widely used to assess depression in Chinese samples (46). The Chinese version of the CES-D was administered to assess parental depression in the current study, and had a Cronbach's alpha of 0.88.

The Parenting Stress Index-Short Form (PSI-SF) is a self-report questionnaire with 36 items that was developed by Abidin (47) with the aim of measuring levels of parenting stress. The PSI-SF has three subscales, including parenting distress (e.g., *feel alone and without friends*), dysfunctional interaction (e.g., *most times feel that child does not like me*), and difficult child (e.g., *my child generally wakes up in a bad mood*), which measure different aspects of parenting stress. Each item is rated based on a five-point Likert scale ranging from “1 = *strongly disagree*” to “5 = *strongly agree*,” with higher scores indicating high levels of parenting stress. The Chinese version of the PSI-SF has good reliability and validity (48), and it was administered in the current study with a Cronbach's alpha of 0.94.

The ISPCAN Child Abuse Screening Tools Parents Version (ICAST-P) is a self-report screening tool developed by Runyan et al. (49) that has been used to measure child maltreatment across nations and cultures (50). Chen et al. (51) translated and validated the ICAST-P to the Chinese cultural context, and the Chinese version of the ICAST-P has 40 items and acceptable reliability and validity. The Chinese version of the ICAST-P has 5 subscales, including moderate physical discipline (12 items; e.g., *Shook him/her aggressively*), severe physical discipline (5 items; e.g., *Burned him/her*), emotional discipline (12 items; e.g., *Shouted at him/her*), neglect (5 items; e.g., *Your child was not given food to eat*), and non-violent discipline (6 items; e.g., *Explained to him/her why something s/he did was wrong*). Participants respond to each item *via* a six-point scale (0 = *never and not in the past year*; 1 = *once or twice a year*; 2 = *several times a year*; 3 = *about once a month*; 4 = *several times a month*; and 5 = *once a week or more often*), and high scores indicate high levels of child maltreatment. The Chinese version of the ICAST-P was administered in the current study to measure child maltreatment, and had a Cronbach's alpha of 0.90.

The Strengths and Difficulties Questionnaire (SDQ) is a self-report questionnaire with 25 items that was developed by Goodman (52) with the aim of measuring the mental strengths manifested in children and the difficulties faced by the children. It has five subscales, including subscales of emotional symptoms (e.g., *Many worries, often seems worried*), conduct problems (e.g., *Often lies or cheats*), hyperactivity (e.g., *Thinking things out before acting*), peer problems (e.g., *Rather solitary, tends to play alone*), and prosocial scale (e.g., *Kind to younger children*). Participants respond to each item *via* a three-point scale (1 = “not true,” 2 = “somewhat true,” and 3 = “certainly true”), and high scores indicate high levels of mental strengths and difficulties. The Chinese version of the SDQ has good reliability and validity (53, 54), and subscales of emotional symptoms, conduct problems, hyperactivity, and peer problems were administered to measure internalizing and externalizing problems of children in the current study, and the Cronbach's alpha of the total four subscales was 0.71.

### Procedure

The authors presented the aims and processes of this study to five preschool headmasters and received their permission to conduct the current study in their preschools. A total of 855 parents from different families signed the informed consent at the beginning of the data collection process and finished the questionnaire booklets within 15 min in the classrooms. Teachers and parents who participated in the study received a small gift ($1), and parents also later received feedback that delineated the current development situation of their child. Concerning the feedback, it did not contain variables of this study, which may not affect the results of this study. The study was approved by the ethics committee of the author's institution, and the procedures and measures of the current study were safe for participants.

### Data analysis

The current study used several steps to perform the data analysis. First, the author cleaned and prepared the data. For example, the author cleaned the data by removing the outliers (25/900) who completed the questionnaires with the same answer except for the demographic questionnaires, and participants (20/900) who finished the questionnaire in < 350 seconds and provided wrong answers for all detecting questions (e.g., please choose “3”). Then normality was examined, and the average scores were computed for each scale, and prepared for the next steps.

Second, the Pearson correlation was conducted to analyze the correlations between variables, and also to examine whether the mediation analysis was suitable for further analysis or not. The data is considered suitable for mediation analysis if the coefficients of pairwise correlations are significant among study variables (55).

Third, mediation analysis is a method to explore the roles of one or more than one variable in relationships between other two variables, which may explain the pathways or mechanisms underlying variables. The current study used mediation analysis to explore the relationships between parenting depression, child internalizing and externalizing problems, parenting stress, and child maltreatment. A direct model was conducted to delineate the relationships between parental depression and child internalizing and externalizing problems, then a serial mediation model was conducted to delineate the roles of parenting stress and child maltreatment in the relationships between parental depression and child internalizing and externalizing problems. All statistical analyses were conducted by R language version 4.1.2 with bruceR packages. A PROCESS (model 6) function with maximum likelihood estimation was used to explore the serial mediation model. Moreover, the Bias-corrected percentile bootstrap method was used to examine the 95% confidence interval (CI) of mediation effects of 1,000 samples. If the 95% CI of the indirect effect contains 0 it means that the indirect effect exists, and if the 95% of the direct effect contains 0 it means that the variable plays a full mediation role in the study variables. All tests were two-tailed for significance, and the *p*-value was set at 0.05. Additionally, the current study used digital questionnaires to collect data, and there were no missing data for all variables.

## Results

### Descriptive statistics

The results of descriptive statistics and correlations between study variables are presented in Table 1. As indicated, parental depression was positively associated with child maltreatment (*r* = 0.14, *p* < 0.01), parenting stress (*r* = 0.34, *p* < 0.01), and internalizing and externalizing problems in children (*r* = 0.14, *p* < 0.01). Parenting stress was positively associated with child maltreatment (*r* = 0.20, *p* < 0.01) and internalizing and externalizing problems in children (*r* = 0.14, *p* < 0.01).

**Table 1:** Means, standard deviations, and correlations between study variables (*n* = 855).

**Variables**	**M**	**SD**	**1**	**2**	**3**	**4**
1 DE	1.45	0.51	–			
2 PS	2.24	0.68	0.34^**^	–		
3 ICAST	1.41	0.85	0.14^**^	0.20^**^	–	
4 IEP	1.50	0.47	0.14^**^	0.14^**^	0.10^**^	–

*DE, Parental depression; PS, Parenting stress; ICAST, Child maltreatment; IEP, Internalizing and externalizing problems*.

** *p*<*0.01*.

### The relationships between parental depression and child internalizing and externalizing problems

The results of the regression analysis are presented in Table 2. The results showed that parental depression was positively associated with child internalizing and externalizing problems (β = 0.127, S.E.= 0.031, *R*^2^ = 0.035, and *p* < 0.001), with controlling children's genders and ages, the number of children in the family, and family monthly income.

**Table 2:** The regression between parental depression, parenting stress, child maltreatment, and internalizing and externalizing problems (*n* = 855).

	**IEP**	**PS**	**ICAST**	**IEP**
Intercept	1.500 (0.016)^***^	2.235 (0.022)^***^	0.9291 (0.105)^***^	1.500 (0.016)^***^
Child gender	0.010 (0.032)	0.040 (0.043)	−0.103 (0.057)	0.012 (0.032)
Number of children	0.049 (0.034)	0.025 (0.046)	0.064 (0.061)	0.045 (0.034)
Child age	−0.003 (0.019)	0.038 (0.026)	−0.016 (0.034)	−0.004 (0.019)
Income	−0.062 (0.021)^**^	−0.170 (0.028)^***^	0.018 (0.038)	−0.054 (0.021)
DE	0.127 (0.031)^***^	0.441 (0.043)^***^	0.131 (0.060)^*^	0.098 (0.033)^**^
PS			0.214 (0.045)^***^	0.047 (0.025)
ICAST				0.037 (0.019)
*R* ^2^	0.035	0.156	0.050	0.044
Adjust *R*^2^	0.029	0.151	0.043	0.036

*DE, Parental depression; PS, Parenting stress; ICAST, Child maltreatment; IEP, Internalizing and externalizing problems;*

* *p*<*0.05;*

** *p*<*0.01;*

*** *p*<*0.001*.

### Mediation analysis

**Figure 2:**
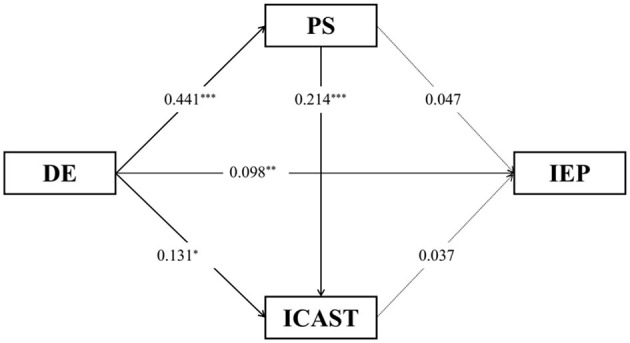
The mediation model between parental depression and children's internalizing and externalizing problems through parenting stress and child maltreatment.

The results of the mediation analysis are presented in Table 3 and Figure 2. The results showed that parental depression was positively associated with parenting stress (β = 0.441, S.E.= 0.043, *R*^2^ = 0.156, and *p* < 0.001) when controlling children's genders and ages, the number of children in the family, and family monthly income. In the model of CES-D-PS-ICAST, parental depression was positively associated with child maltreatment (β = 0.131, S.E.= 0.060, *R*^2^ = 0.050, and *p* < 0.05), and parenting stress was positively associated with child maltreatment (β = 0.241, S.E.= 0.045, *R*^2^ = 0.050, and *p* < 0.001). In the model of CES-D-PS-ICAST-IEP, parental depression was positively associated with child internalizing and externalizing problems (β = 0.098, S.E.= 0.033, and *p* < 0.01) when controlling children's genders and ages, the number of children in the family, and family monthly income. Moreover, the results of the Bias-corrected percentile method showed that the 95% CI of indirect effects of parenting stress and child maltreatment were [−0.058, 0.043] and [0.000, 0.019], respectively, and the 95% CI of direct effect was [0.030, 0.160], which suggested that child maltreatment mediated the relationships between parental depression and children's internalizing and externalizing problems. Additionally, the results of the Bias-corrected percentile method showed that the 95% CI of all indirect effects of the combination of parenting stress and child maltreatment was [0.000, 0.160], which suggested that parenting stress and child maltreatment mediated the relationships between parental depression and child internalizing and externalizing problems. Additionally, a moderation analysis was also performed, and the results showed that there were no gender effects.

**Table 3:** The indirect paths between parental depression and internalizing and externalizing problems through parenting stress and child maltreatment (*n* = 855).

	**Estimate**	**S.E**.	**Z**	***P***	**[Boot 95% CI]**	**β**
Indirect_All	0.029	0.012	2.408	< 0.016^*^	[0.005, 0.051]	0.031
Ind_X_M1_Y	0.021	0.012	1.700	< 0.089	[−0.058, 0.043]	0.022
Ind_X_M2_Y	0.005	0.004	1.146	0.252	[0.000, 0.019]	0.005
Ind_X_M1_M2_Y	0.003	0.002	1.557	0.119	[0.000, 0.009]	0.004
Direct	0.098	0.034	2.889	0.004^**^	[0.030, 0.160]	0.106
Total	0.127	0.031	4.069	< 0.001^***^	[0.065, 0.184]	0.137

*X, parental depression; Y, internalizing and externalizing problems; M1, parenting stress; M2, child maltreatment;*

* *p*<*0.05;*

** *p*<*0.01;*

*** *p*<*0.001*.

## Discussion

The present study explored the relationships between parental depression and child internalizing and externalizing problems and examined the roles of parenting stress and child maltreatment in those relationships within the Chinese cultural context. As such, it broadens the scopes of childhood and family studies. These findings suggested that parental depression was positively associated with child internalizing and externalizing problems, and child maltreatment and the combination of parenting stress and child maltreatment mediated the relationships between parental depression and child internalizing and externalizing problems, respectively.

The results showed that parental depression was positively associated with parenting stress, which was consistent with previous studies (56). Individuals with depression may have less self-efficacy (57), which may contribute to high levels of parenting stress. The results also showed that parental depression was positively associated with child maltreatment, which was consistent with previous studies (58, 59). Similarly, according to the family system theory, depressed parents may impair child development by causing internalizing and externalizing problems in their children. Meanwhile, parenting stress was positively associated with child maltreatment, which was consistent with previous studies (41). These findings suggest that parental depression may be an important factor in child development.

Moreover, the results showed that child maltreatment mediated the relationships between parental depression and child internalizing and externalizing problems, which indicated that parental depression not only had a direct effect on child internalizing and externalizing problems but also had an indirect effect *via* child maltreatment. Individuals with high levels of depression may have disorganized interactions with children, and these disorganized interactions may be considered as some kind of child maltreatment, contributing to child internalizing and externalizing problems (60). Depressed parents may reject their children, which may increase child internalizing and externalizing problems (61). These findings suggest that child maltreatment may be one of the bridges between parental depression and child internalizing and externalizing problems.

Additionally, the results showed that parenting stress and child maltreatment progressively mediated the relationships between parental depression and child internalizing and externalizing problems, which suggested that parental depression influenced child internalizing and externalizing problems *via* the combination of parenting stress and child maltreatment. Individuals with depression may have low levels of self-efficacy (57) and attention biases in negative information (29), which may contribute to high levels of parenting stress, and these high levels of parenting stress may increase the risk of child maltreatment, which, in turn, contributes to high levels of child internalizing and externalizing problems. The results confirm the assumptions offered by the model of depressed mothers and maladaptation of children and those proposed by the family system theory, and raises the importance of exploring parental depression. These findings suggest that parental depression influences how parents treat their children (e.g., parenting stress and child maltreatment) and that it can lead to child internalizing and externalizing problems.

Some conclusions can be reached with the results of the current study. First, parental mental health is an important issue for children and families, and may increase the risk of parenting stress, child maltreatment, and child internalizing and externalizing problems. Governments and communities should invest in some programs that aim to improve the mental health of parents, and provide some strategies for preserving positive mental health. Second, parenting stress and child maltreatment are two mediators in the relationships between parental depression and child internalizing and externalizing problems, and decreasing parenting stress and child maltreatment may reduce the influence of parental depression on child internalizing and externalizing problems. Governments and communities should support parents with materials and mental health support through some programs, which may decrease parenting stress. Parents should learn some positive strategies for educating children, which may decrease the risk of child maltreatment.

Some limitations should be acknowledged in the current study. First, the present study contained few male participants who were parents, which may affect the data on parental depression. Future studies should recruit couples for the study, which may give much more solid evidence on parental depression and parenting stress. Second, the current study used cross-sectional designs to examine the pathways between parental depression and child internalizing and externalizing problems, which may not verify a causal relationship. Future studies should apply longitudinal designs to examine the mechanisms linking parental depression and child internalizing and externalizing problems. Third, the current study used self-report questionnaires to collect the data, which may lead to results that incorporated memory bias and social desirability. Future studies should collect data with different methods (e.g., interviews, questionnaires, and experiments), which may provide much more accurate information.

## Conclusion

The present study explored the relationships between parental depression and child internalizing and externalizing problems, and examined the roles of parenting stress and child maltreatment in those relationships within the Chinese cultural context. In doing so, it broadens the scopes of family studies. The findings suggested that parental depression was positively associated with child internalizing and externalizing problems, and child maltreatment and that the combination of parenting stress and child maltreatment mediated the relationships between parental depression and child internalizing and externalizing problems, respectively. Parental depression not only had a direct effect on child internalizing and externalizing problems but also had an indirect effect *via* parenting stress and child maltreatment. Decreasing the levels of parenting stress and child maltreatment should be applied in interventions to break the relationships between parental depression and child internalizing and externalizing problems in the Chinese cultural context.

## Data availability statement

The original contributions presented in the study are included in the article/Supplementary material, further inquiries can be directed to the corresponding author.

## Ethics statement

The studies involving human participants were reviewed and approved by Beijing Normal University. The patients/participants provided their online informed consent to participate in this study.

## Author contributions

CC designed the study, written and revised the manuscript, and completed the data analysis.
